# Brain Injury and Bad Temper

**Published:** 1889-01-05

**Authors:** 


### BRAIN INJURY AND BAD TEMPER.

At a well-known medical society, a boy was recently shown whose case is worth thinking of by hasty mothers, schoolmasters who strike children on the head, and others. On April 16th a window-pole was pushed through the boy's skull into the brain substance, for the depth of about an inch. The point of the pole entered the upper and back part of the head. The accident gave rise to fever and some delirium. It also produced "bad temper." Many people, perhaps the majority, consider that "bad temper" is entirely voluntary on the part of the person who displays it. As a matter of fact, it is often to a very great extent involuntary, and no one is more angry at it than the bad-tempered person himself. Of course, everyone, whether he is born with a bad temper, or has acquired one from habit, or has been visited with one as the result of disease or injury, should at least try to control it. But his friends should also bear in mind that bad temper may be, and often is, an affliction to be sympathised with, not an offence to be punished. This boy, whose head was pierced with a window-pole on April 16th, remained bad-tempered until July 20th, and then an abscess of the brain broke through the wound. All that time the poor little fellow had been enduring the pain and distress of a gradually-forming abscess. The abscess was treated by antiseptics in the ordinary way. The patient's other symptoms were soothed and relieved by appropriate treatment, and on September 5th he was able to leave the hospital, not only quite well in himself, but with his bad temper as completely cured as his other disordered symptoms.

